# Dibutyltin Disrupts Glucocorticoid Receptor Function and Impairs Glucocorticoid-Induced Suppression of Cytokine Production

**DOI:** 10.1371/journal.pone.0003545

**Published:** 2008-10-28

**Authors:** Christel Gumy, Charlie Chandsawangbhuwana, Anna A. Dzyakanchuk, Denise V. Kratschmar, Michael E. Baker, Alex Odermatt

**Affiliations:** 1 Division of Molecular and Systems Toxicology, Department of Pharmaceutical Sciences, University of Basel, Basel, Switzerland; 2 Department of Nephrology and Hypertension, University of Berne, Berne, Switzerland; 3 Department of Medicine, University of California San Diego, La Jolla, California, United States of America; Illinois Institute of Technology, United States of America

## Abstract

**Background:**

Organotins are highly toxic and widely distributed environmental chemicals. Dibutyltin (DBT) is used as stabilizer in the production of polyvinyl chloride plastics, and it is also the major metabolite formed from tributyltin (TBT) *in vivo*. DBT is immunotoxic, however, the responsible targets remain to be defined. Due to the importance of glucocorticoids in immune-modulation, we investigated whether DBT could interfere with glucocorticoid receptor (GR) function.

**Methodology:**

We used HEK-293 cells transiently transfected with human GR as well as rat H4IIE hepatoma cells and native human macrophages and human THP-1 macrophages expressing endogenous receptor to study organotin effects on GR function. Docking of organotins was used to investigate the binding mechanism.

**Principal Findings:**

We found that nanomolar concentrations of DBT, but not other organotins tested, inhibit ligand binding to GR and its transcriptional activity. Docking analysis indicated that DBT inhibits GR activation allosterically by inserting into a site close to the steroid-binding pocket, which disrupts a key interaction between the A-ring of the glucocorticoid and the GR. DBT inhibited glucocorticoid-induced expression of phosphoenolpyruvate carboxykinase (PEPCK) and tyrosine-aminotransferase (TAT) and abolished the glucocorticoid-mediated transrepression of TNF-α-induced NF-κB activity. Moreover, DBT abrogated the glucocorticoid-mediated suppression of interleukin-6 (IL-6) and TNF-α production in lipopolysaccharide (LPS)-stimulated native human macrophages and human THP-1 macrophages.

**Conclusions:**

DBT inhibits ligand binding to GR and subsequent activation of the receptor. By blocking GR activation, DBT may disturb metabolic functions and modulation of the immune system, providing an explanation for some of the toxic effects of this organotin.

## Introduction

Organotins are among the most toxic and widely distributed environmental chemicals. The most abundant organotin in the environment is tributyltin (TBT), a molluscicide and fungicide widely used as an antifouling paint for boat and fish nets and that thus is dispersed into the marine environment [1], [2]. TBT interferes with reproduction in marine animals, inducing imposex (superimposition of male sexual characters in females) in gastropod molluscs, an effect used to measure TBT pollution in sea-water [3], [4]. TBT concentrations ranging from 4–323 nM were measured in blood samples from healthy human subjects [5]. In mammals, organotins are hepatotoxic, neurotoxic and immunotoxic. At doses comparable with those found in human blood, TBT promotes Th2 cell polarization and exacerbates airway inflammation, providing a possible mechanism for enhanced susceptibility to allergic diseases [6]. Thus, TBT contamination represents a serious health problem and the great concern about the toxicity of TBT is underlined by recent negotiations of the United Nations' International Maritime Organization for a global ban in the use of TBT.

*In vivo*, TBT is mainly metabolized to DBT in the liver, involving cytochrome P450 enzymes [7], [8]. DBT, itself, is used in the production of polyvinyl chloride (PVC) plastic tubes and bottles [9], and humans are exposed to DBT by direct uptake from drinking water due to leaching from PVC water distribution pipes [10]. DBT is considered to be highly neurotoxic and immunotoxic [5], [11], [12], and concentrations ranging from 11–401 nM were measured in human blood [5]. Hence, DBT needs to be considered as a potential toxic chemical. Both TBT and DBT cause thymus involution by inhibiting the proliferation of immature CD4^−^/CD8^+^ thymocytes, but at high concentrations, they induce thymocyte apoptosis [13]. The immunotoxic effects of DBT are more rapid and more pronounced than those of TBT, raising the possibility that some effects of TBT are primarily caused by its metabolite DBT [14]. The target(s) for the immunotoxic actions of DBT have not been determined.

We hypothesize that DBT affects one or more glucocorticoid responses because this steroid has important actions on the immune system [15], [16]. Despite the importance of glucocorticoids in the regulation of immune functions, only few studies investigated the potential interference of environmental pollutants with glucocorticoid-mediated responses [17]. None of these studies investigated DBT as a disruptor of the immune response.

With the goal of elucidating the immunotoxic mechanism of DBT and other organotins, we investigated their potential interference with glucocorticoid action using cells expressing recombinant GR, as well as macrophages expressing endogenous GR. We found that DBT, in contrast to TBT, diphenyltin (DPT) and triphenyltin (TPT), inhibits dexamethasone binding to GR and blocks receptor activation. We then used docking software [18], [19] to study the binding of these chemicals to the GR and provide evidence that DBT, but not the other organotins, disrupts an essential interaction between the A-ring on dexamethasone and the GR by binding to an allosteric site.

## Results

### Dibutyltin, but not other organotins tested, inhibits the transcriptional activity of GR

**Figure 1:**
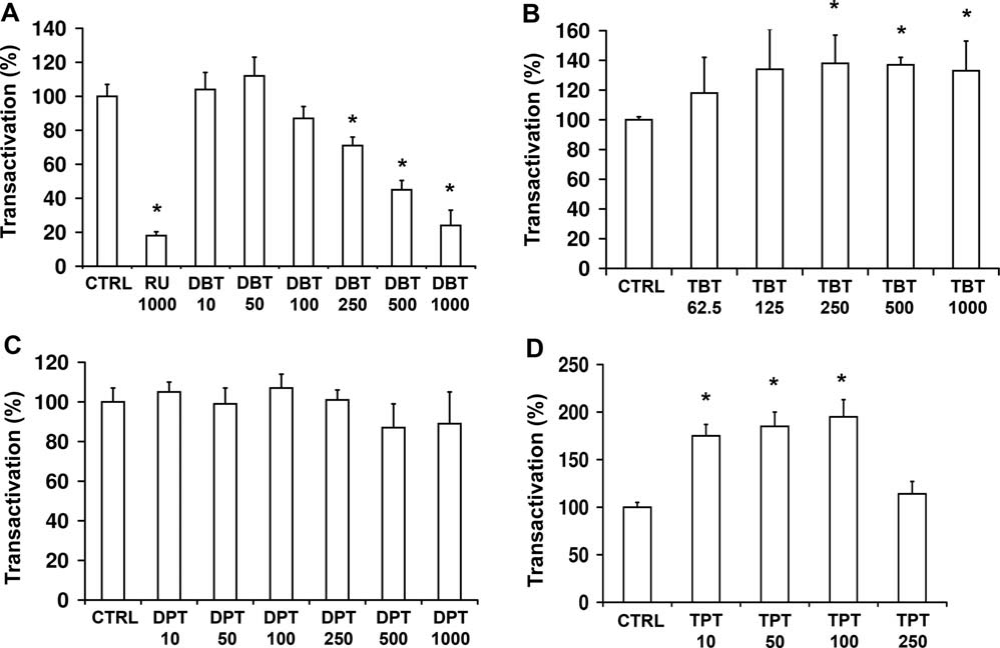
Inhibition of GR-mediated transactivation by organotins. HEK-293 cells transiently expressing pMMTV-LacZ, pCMV-LUC and human GR-α were simultaneously incubated with 100 nM cortisol and vehicle (CTRL), various concentrations of dibutyltin (DBT) (*A*), tributyltin (TBT) (*B*), diphenyltin (DPT) (*C*), triphenyltin (TPT) (*D*) or 1 µM of RU486 (RU). After incubation for 20 h, galactosidase reporter activity, normalized to the internal luciferase control, was determined. Data (mean±SD from four independent experiments) represent percentage relative to the control at 100 nM cortisol in the absence of inhibitors. *p<0.05.

To investigate whether organotins disrupt GR-dependent transcriptional regulation, we employed HEK-293 cells transiently expressing recombinant human GRα and a β-galactosidase reporter driven by a glucocorticoid-responsive MMTV-promoter. In the absence of organotins, 100 nM cortisol increased GR-dependent expression of galactosidase activity 185-fold. HEK-293 cells that were transfected only with the β-galactosidase reporter showed background activity because HEK-293 cells express no or very low levels of endogenous GR. Upon simultaneous incubation of cells with DBT and cortisol, a dose-dependent inhibition of GR-mediated transactivation was observed (Fig. 1A). At 1 µM DBT, the inhibition was comparable with that of 1 µM RU486. Interestingly, the repeated exposure of cells to DBT increased the inhibitory effect on GR-mediated transactivation. Upon subjecting cells to eight changes of medium containing a given DBT concentration at 15 min intervals and preincubation for another 14 h, a two- to three-fold stronger inhibition of GR transactivation was observed than in cells that were preincubated for 16 h with the same DBT concentration (data not shown). In contrast to DBT, a slight stimulation of transactivation was found for TBT, reaching a maximal effect at 250 nM (Fig. 1B). Whereas DPT had no effect on GR-mediated transactivation (Fig. 1C), an up to two-fold stimulation was observed for TPT, with a 75% increase of transactivation at 10 nM (Fig. 1D). This stimulation was reversed at 250–500 nM. At these concentrations, no cytotoxicity could be detected in the MTT assay. Dimethyltin, trimethlytin and dioctyltin, up to 1 µM, did not affect GR-mediated transactivation. Similar results were obtained in experiments using 10 nM dexamethasone instead of 100 nM cortisol. None of the organotins tested stimulated GR-mediated transactivation in the absence of glucocorticoids (data not shown).

### Dibutyltin blocks ligand binding to GR

**Figure 2:**
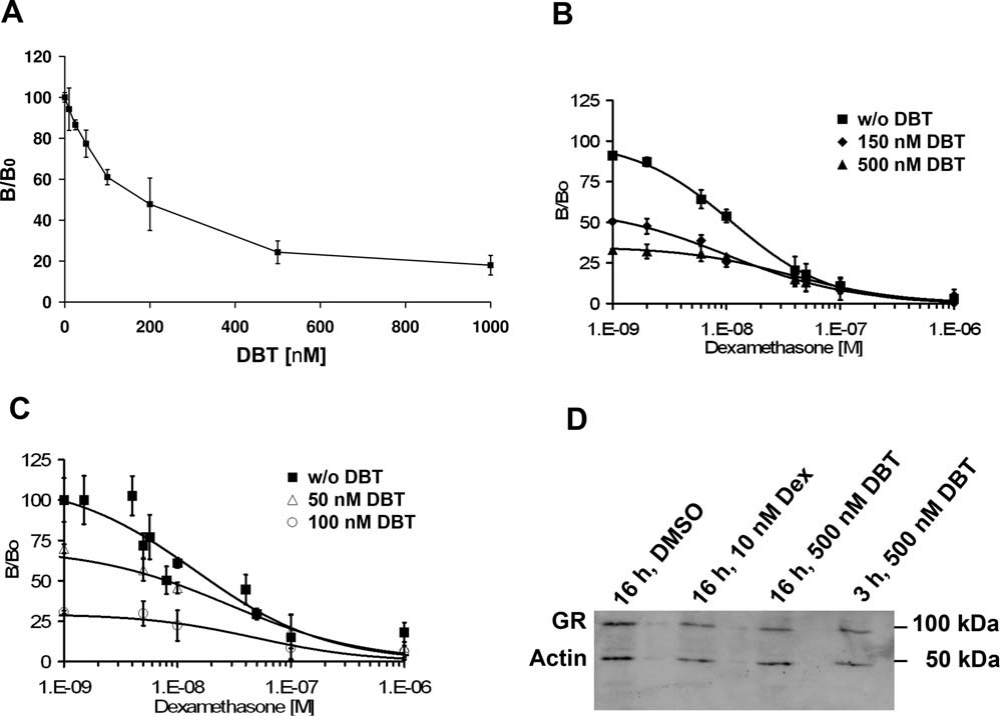
Inhibition of ligand binding to GR by dibutyltin. HEK-293 cells were transfected with a plasmid for human GR-α, followed by overnight incubation in serum-free medium containing vehicle (0.1% DMSO)(*A*,*B*) or in medium containing either vehicle or 50 nM or 100 nM DBT (*C*). Cells were then incubated for 3 h with 10 nM radiolabeled dexamethasone and various concentrations of DBT (*A*) or with 10 nM radiolabeled dexamethasone, various concentrations of unlabeled dexamethasone and various concentrations of DBT (*B*,*C*) prior to removal of unbound steroids and determination of bound dexamethasone by scintillation counting (*A*). Data represent mean±SD from three independent experiments. *D*, cells transiently expressing GR-α were preincubated for 16 h in serum-free medium containing vehicle (0.1% DMSO), 10 nM dexamethasone (Dex) or 500 nM DBT, followed by incubation for another 3 h with vehicle, 10 nM dexamethasone or 500 nM DBT and 10 nM dexamethasone, respectively (lane 1–3). Alternatively, cells preincubated for 16 h in serum-free medium were incubated for 3 h in medium containing 500 nM DBT and 10 nM dexamethasone (lane 4). Cells were lysed and the expression of GR protein and actin control was analyzed by Western blotting.

To assess whether DBT inhibits GR function by directly interfering with ligand binding, HEK-293 cells were simultaneously incubated with radiolabeled [^3^H]-dexamethasone and various concentrations of DBT. A dose-dependent decrease of dexamethasone binding to GR was observed, with approximately 50% inhibition of binding upon incubation with 150 nM DBT (Fig. 2A). Addition of unlabeled dexamethasone revealed a typical displacement pattern of tritiated dexamethasone in the presence or absence of DBT (Fig. 2B,C). A more pronounced inhibitory effect by DBT on dexamethasone binding to GR was obtained after preincubating cells overnight with DBT-containing medium, with an IC_50_ of about 50 nM (Fig. 2C). To determine if the reduced dexamethasone binding to GR upon preincubation with DBT was due to decreased GR protein expression, we performed Western blotting of extracts from cells expressing GR and preincubated with either vehicle or DBT. Incubation with DBT for either 3 h or 16 h did not alter GR expression relative to the actin control (Fig. 2D), suggesting that DBT did not affect the number of receptor complexes but decreased glucocorticoid binding affinity. Thus, inhibition of glucocorticoid binding to GR in the presence of DBT provides an explanation for the reduced transactivation of the β-galactosidase reporter.

### DBT disrupts glucocorticoid-dependent target gene regulation in liver cells

**Figure 3:**
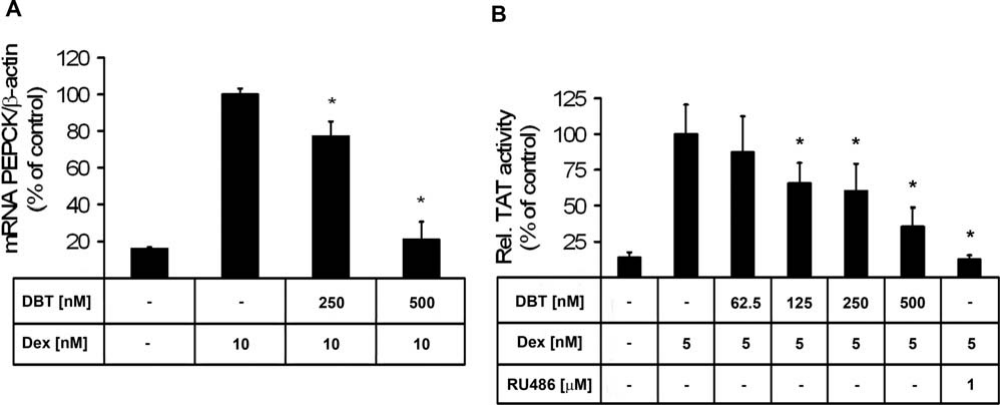
Inhibition of glucocorticoid-mediated expression of hepatic target genes by dibutyltin. *A*, DBT prevents glucocorticoid-induced stimulation of PEPCK mRNA expression. H4IIE hepatoma cells were preincubated for 16 h in the absence or presence of DBT, followed by adding 10 nM dexamethasone and incubation for another 24 h at 37°C. mRNA levels were determined by real-time RT-PCR using TaqMan technology. Data (mean±S.D. of triplicates from four independent experiments) are relative to the ratio of PEPCK mRNA to β-actin control mRNA from cells treated with dexamethasone in the absence of DBT. *B*, DBT inhibits glucocorticoid-induced stimulation of TAT activity. H4IIE cells were preincubated for 16 h in the absence or presence of DBT, followed by adding 5 nM dexamethasone and incubation for another 20 h. TAT activity, determined in cell lysates and normalized to protein content, is given as mean±S.D. of triplicates from four independent experiments and normalized to the positive control in the presence of dexamethasone. * p<0.05.

The observation that DBT blocked GR activation in an overexpression cell system led us to test whether the disruption of GR activation could be observed in cells expressing endogenous receptor. Incubation of rat H4IIE hepatoma cells, a widely used liver cell model, with 10 nM dexamethasone for 24 h resulted in elevated PEPCK mRNA expression approximately 6-fold (Fig. 3A). This glucocorticoid-induced stimulation of PEPCK mRNA expression was diminished in a dose-dependent manner when cells were preincubated for 16 h with DBT. The effect of glucocorticoids was almost completely abolished in the presence of 500 nM DBT.

As a second marker of GR activation, we measured the effect of DBT on TAT activity. Incubation with 5 nM dexamethasone increased TAT activity approximately 6-fold in H4IIE hepatoma cells. (Fig. 3B). Treatment of cells with DBT dose-dependently diminished the dexamethasone-induced stimulation of TAT activity. GR antagonist RU486 (1 µM) was used as a control. DBT had no effect on basal TAT activity in the absence of glucocorticoids (data not shown).

### DBT impairs glucocorticoid-mediated suppression of cytokine production in macrophages

**Figure 4:**
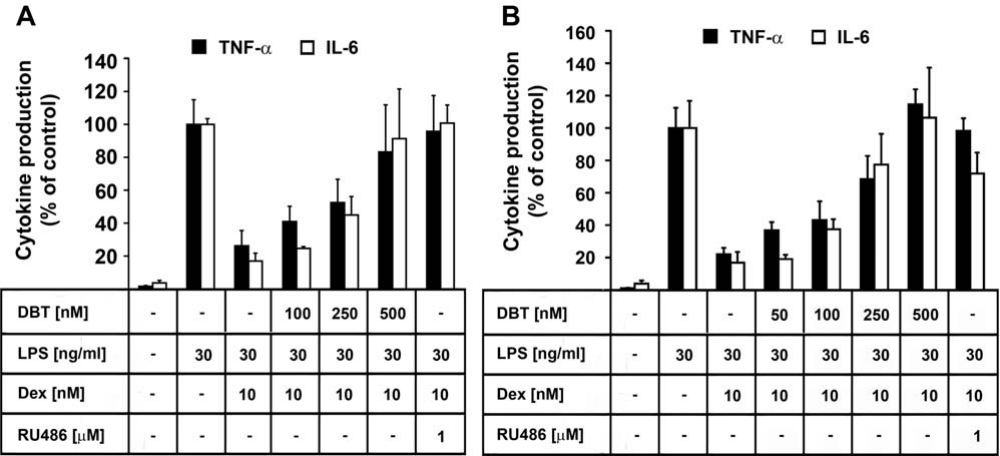
Dibutyltin abrogates GR-mediated suppression of cytokine production. Native human monocytes (*A*) or THP-1 cells (*B*) were differentiated into macrophages, incubated for 16 h with various concentrations of DBT prior to the addition of LPS and dexamethasone. Cells were incubated for another 20 h, and the levels of TNF-α (filled bars) and IL-6 (open bars) were determined by ELISA. Data represent mean±SD from three independent experiments, normalized to the LPS-stimulated control.

Exposure of macrophages to LPS strongly induces production of various pro-inflammatory cytokines. Among them, TNF-α and IL-6 play a pivotal role in the inflammatory response. The anti-inflammatory effect of glucocorticoids is at least in part due to the modulation of cytokine-production in a GR-dependent manner. To assess potential interference of DBT with glucocorticoid-dependent suppression of pro-inflammatory cytokines, native human monocytes were differentiated into macrophages. Macrophages were then preincubated for 16 h with vehicle or DBT, followed by simultaneous addition of LPS and 10 nM dexamethasone and incubation for another 20 h. Detection by ELISA revealed a DBT dose-dependent abrogation of the dexamethasone-mediated suppression of TNF-α and IL-6 production after LPS-induced stimulation (Fig. 4A). At 500 nM the effect of DBT was comparable with that of 1 µM RU486. DBT alone had no effect on IL-6 or TNF-α production (not shown). Similar results were observed in experiments with THP-1 cells that were differentiated into macrophages by treatment with PMA (Fig. 4B).

### Dibutyltin inhibits GR-mediated trans-repression of NF-κB

**Figure 5:**
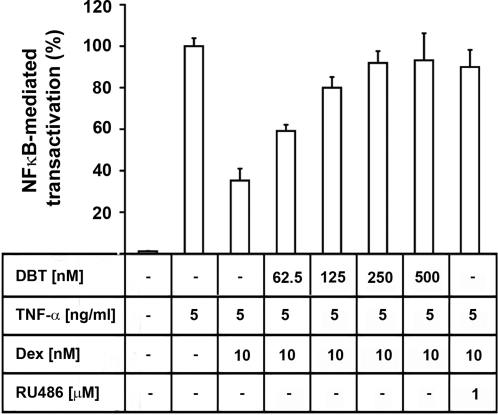
Inhibition of GR-mediated transrepression and potentiation of TNF-α-induced activation of NF-κB. HEK-293 cells were transfected with a plasmid for a luciferase reporter gene under the control of a promoter containing three NF-κB binding sites together with a plasmid for human GR. Cells were preincubated for 16 h with various concentrations of DBT prior to stimulation with TNF-α in the presence of glucocorticoids. Data represent mean±S.D. from three independent experiments, normalized to the TNF-α -stimulated control.

Trans-repression of NF-κB activity by GR is important for the anti-inflammatory action of glucocorticoids. Therefore, we tested whether DBT might reverse glucocorticoid-dependent inhibition of NF-κB activation upon incubation with TNF-α. TNF-α strongly stimulated the expression of a luciferase reporter gene driven by the MHC promoter containing three NF-κB binding sites, an effect which was suppressed by dexamethasone (Fig. 5). As for the cytokine production, the glucocorticoid-mediated suppression of NF-κB activation was inhibited in a dose-dependent manner by DBT or by RU486.

### Docking of organotins into human GR

**Figure 6:**
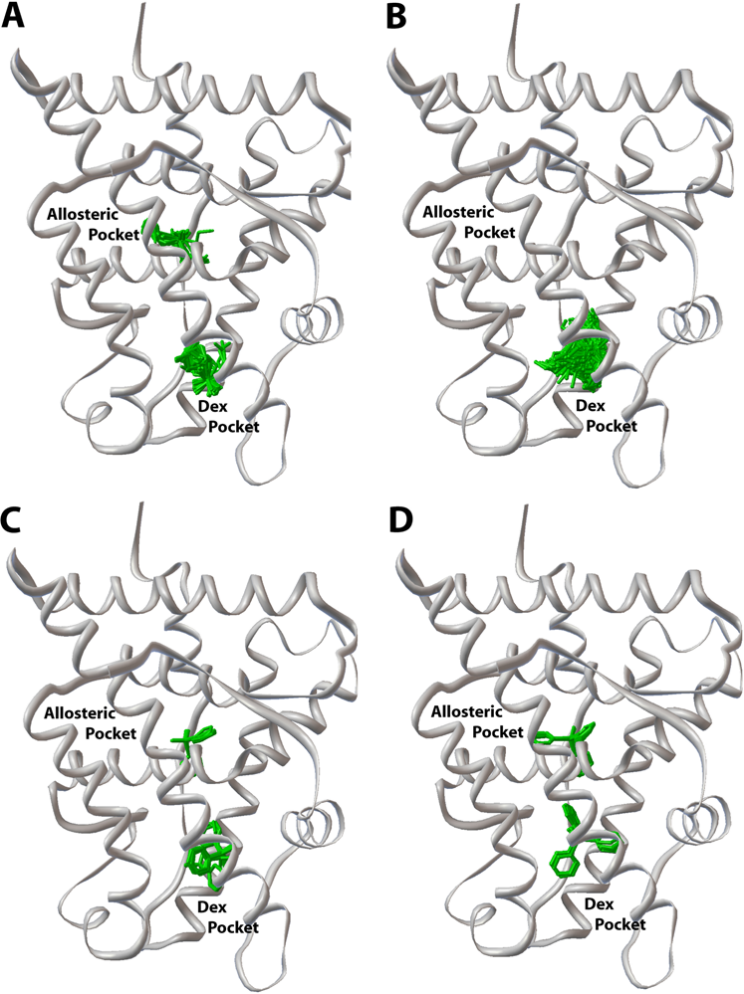
Docking sites for organotins in the GR. Autodock 4 was used to dock organotins (green) into the GR (1M2Z.pdb) (grey) without dexamethasone. DBT (*A*) docked into two sites on GR, with the highest percentage in the dexamethasone-binding site (Table 1). The second site for DBT was an adjacent allosteric pocket. Docking of TBT (*B*), DPT (*C*) and TPT (*D*) into the GR.

To study the mechanism by which DBT inhibits dexamethasone binding to GR, we used Autodock 4 [18], [19] to screen the GR 3D-structure [21] for binding sites for DBT and other organotins. We identified two binding sites for DBT, DPT and TPT and one site for TBT (Fig. 6). These are the steroid-binding site and a site that is adjacent to the steroid-binding site [21]. Differences in the relative occupancy for each organotin in the GR are summarized in Table 1. The dexamethasone binding site was the primary binding site in the GR for all organotins. Occupancy of the steroid-binding site varied from 51% for TPT to 100% for TBT.

**Table 1:** Relative calculated occupancy by organotins in the GR.

Organotin	Dexamethasone pocket	Allosteric pocket
Dibutyltin	82%	18%
Tributyltin	100%	
Diphenyltin	87%	13%
Triphenyltin	56%	44%

In docking earch organotin into the GR, Autodock provides a summation of the van der Waals, hydrogen bond and desolvation energies (summation binding energy) and a separate calculation of the electrostatic binding energy. For organotins, the summation binding energy consists mainly of hydrophobic interactions. In general, the electrostatic binding energy is about 20% to 25% of the summation binding energy for the binding of the organotins into the GR. Thus for DBT, TBT, DPT and TPT in the steroid-binding site, the summation binding energies are −20.1 kJ/mol, −26.1 kJ/mol, −22.4 kJ/mol and −26.7 kJ/mol, respectively. DBT, which is the smallest organotin has the weakest summation binding energy. For DBT, TBT, DPT and TPT the electrostatic binding energies are −3.6 kJ/mol, −2.5 kJ/mol, −1.5 kJ/mol and −0.3 kJ/mol, respectively. Interestingly, DBT has the strongest electrostatic binding energy and TPT has the lowest electrostatic binding energy in the steroid-binding site.

For DBT, DPT and TPT in the allosteric site, the summation binding energies are −18.2 kJ/mol, −22.1 kJ/mol, −28.2 kcal/mol, respectively. For DBT, DPT and TPT, the electrostatic binding energies are −5.1 kJ/mol, −4.8 kJ/mol and −5.0 kJ/mol, respectively.

### Organotin docking to the dexamethasone-binding site

**Figure 7:**
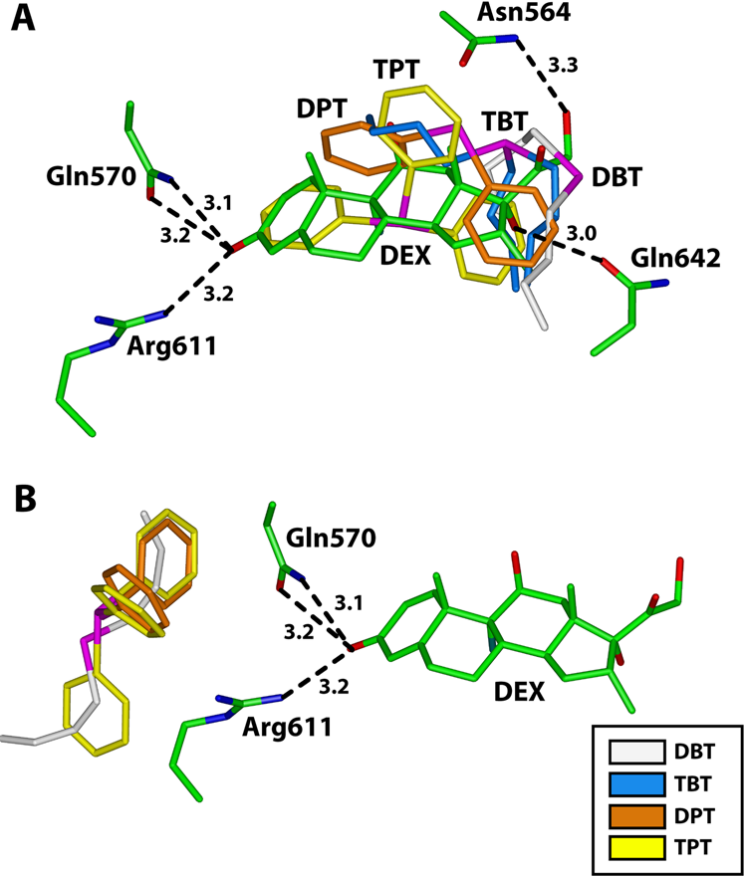
Docking of organotins into the dexamethasone-binding site and at an allosteric site in the GR. *A*, DBT (white), TBT (blue), DPT (orange) and TPT (yellow) fit into the dexamethasone-binding site of the GR. In these unminimized complexes with the GR, DBT and TBT overlap the D-ring and DPT and TPT most of dexamethasone. Distances between dexamethasone and Gln^570^, Arg^611^, Asn^564^ and Gln^642^ are taken from the crystal structure [21]. *B*, organotins docked into the GR at an allosteric site close to Glu^570^ and Arg^611^.

Figure 7A shows DBT, TBT, DPT and TPT in the dexamethasone-binding site of the GR derived from the structure determined by Bledsoe *et al.* [21]. DBT and TBT overlap the region where the D-ring and C17-side chain of dexamethasone insert into the GR. Both DBT and TBT are much smaller than dexamethasone, which reduces interactions of these organotins with the GR. In contrast, DPT and TPT overlap much of the dexamethasone-binding site in the GR. In Figure 7A, we show four residues that are important in stabilizing binding of dexamethasone to the GR [21].

### Organotin docking to an allosteric site

DBT, DPT and TPT docked into the GR at an allosteric site, which is close to the A-ring of dexamethasone (Fig. 7B). Of the four organotins that we studied, only DBT inhibits transcriptional activity of GR in the presence of 100 nM cortisol, which indicates that DBT exerts its inhibitory action by binding to the allosteric site. To investigate further the interaction of GR with DBT in this secondary site, we analyzed the interaction of DBT with GR under two conditions. First, we analyzed DBT in the apo-GR to simulate DBT binding in the absence of glucocorticoids. Second, we analyzed DBT in the GR-dexamethasone complex.

For the first analysis, we docked DBT into the GR and minimized the DBT-GR complex with Discover 3, to allow all of the amino acids in the GR to relax, so as to diminish unfavorable steric interactions and optimize favorable interactions between GR and DBT. Then, we inserted dexamethasone into the minimized structure and analyzed the interactions between DBT and dexamethasone with the GR. In this analysis, we focus on the effect of DBT on the interactions between the C3-ketone on dexamethasone and Arg^611^ and Gln^570^ in the GR because these amino acids have been shown to be important in stabilizing the A-ring of dexamethasone [21]. We also examined the interaction of Gln^642^ and Asn^564^ with substituents on the D ring of dexamethasone.

**Figure 8:**
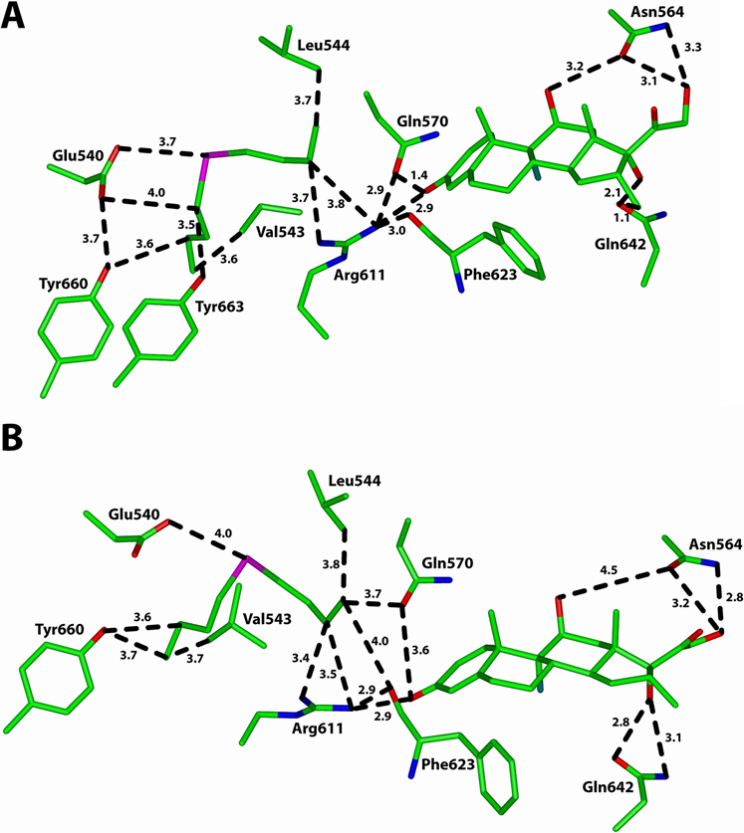
Interactions of dibutyltin with the GR in an allosteric site close to the dexamethasone-binding site. *A*, After minimization of the 3D-model of the DBT-GR complex, dexamethasone was inserted into the complex. There are steric clashes between dexamethasone and Gln^570^ and Gln^642^, which are absent in the GR crystal structure [21] (Fig. 7A). The distances of Asn^564^, Arg^611^ and Phe^623^ to dexamethasone are similar to that in the GR crystal structure. *B*, Interaction of dexamethasone with the GR after energy minimization of the DBT-GR-dexamethasone complex in (*A*) with Discover 3. Gln^570^ and Gln^642^ have moved and no longer have steric clashes with dexamethasone. Asn^564^, however, has moved to 4.5 Å from the C11 hydroxyl on dexamethasone.

Analysis of DBT in the allosteric site of the GR reveals van der Waals contacts between DBT and Tyr^660^, Tyr^663^, Val^543^, Leu^544^ and Arg^611^. There also is a coulombic interaction between Glu^540^ and tin. Importantly, as shown in Figure 8A, there are two critical steric clashes between dexamethasone and the GR in the minimized GR-DBT complex. First, Gln^570^ is 1.4 Å from the C3-ketone, and second, Gln^642^ is 1.1 Å from the C17 hydroxyl group. Interestingly, other key amino acids, such as Asn^564^, Arg^611^ and Phe^623^ [21], retain their favorable binding to dexamethasone. Thus, the 3D-models indicate that binding of DBT to an allosteric site in unliganded GR selectively distorts the ligand-binding domain and alters the interaction with dexamethasone.

To determine if the ternary DBT-GR-dexamethasone complex could relax into a conformation that would be favorable for dexamethasone binding, we minimized this complex using Discover 3. Analysis of this complex (Fig. 8B) reveals that after energy minimization the steric clashes between dexamethasone and Gln^570^ and Gln^642^ have been removed. After minimization, Gln^642^ moved to have a strong hydrogen bond with the C17 hydroxyl group, which also is present in the GR crystal structure [21]. Gln^570^ is more distant from the C3 ketone than in the crystal structure (Fig 7A). This change may be due to a van der Waals contact between Gln^570^ and DBT. Figure 8B also shows that there are changes in other interactions between DBT and residues in the allosteric pocket of the GR.

An important change, which has profound functional implications for steroid activation of the GR, is the loss of the contact between Asn^564^ and the C11 hydroxyl on dexamethasone. Asn^564^ and corresponding asparagines on the progesterone receptor (PR) and mineralocorticoid receptor (MR) are important in stabilizing binding and transcriptional activity of cognate steroids to these receptors [22]. Asn^564^ and Gln^570^ are on α-helix 3 on the GR, PR and MR. Arg^611^ is on α-helix 5. The distance between α-helix 3 and α-helix 5 is crucial for steroid activation of the GR, PR [22] and MR [23]. Our analysis indicates that DBT will alter the conformation of α-helix 3 in the GR-dexamethasone complex.

## Discussion

Despite the fact that organotins are well known and relevant environmental pollutants, we are only beginning to understand their mechanisms of toxicity. An important advance was the recent observation that the triorganotins TBT and TPT are potent activators of the nuclear hormone receptors RXR and PPARγ and that they promote adipocyte differentiation, suggesting that these organotins might contribute to the development of metabolic diseases [24]–[27]. Interestingly, the diorganotins DBT and DPT were found to be inactive toward RXR and PPARγ in these studies.

The pathophysiological effects of DBT have been less well studied and understood. Here, we demonstrate for the first time that DBT at nanomolar concentrations, but not other organotins investigated, disrupts GR function. In a recent study we showed that the organotins DBT, TBT, DPT and TPT inhibited the enzyme 11β-HSD2, which catalyzes the conversion of active 11β-hydroxyglucocorticoids into their inactive 11-keto derivatives [28]. IC_50_ values of 11β-HSD2 inhibition between 1 µM for TPT and 5 µM for DBT were obtained. Thus, while some organotins such as TPT may enhance local glucocorticoid effects by inhibiting 11β-HSD2-dependent glucocorticoid inactivation, DBT blocks GR activation at concentrations that are ten-fold lower than those effective on 11β-HSD2. Importantly, 11β-HSD2 is not expressed in HEK-293 and H4EII cells as well as THP-1 and native human macrophages that are used in the present study, and the observed effects of DBT are not caused by altered intracellular glucocorticoid metabolism. These observations suggest that different organotins cause distinct disturbances of corticosteroid hormone action *in vivo*.

The ability of DBT to inhibit GR function is relevant for human toxicity from two ways. First, humans are exposed to DBT through its use as a heat stabilizer in the production of PVC plastic materials (water distribution pipes, tubings and bottles) from which it can leach into drinking water [9]. And second, DBT is likely to be responsible for some of the toxicity of TBT in the environment, because earlier *in vivo* studies indicated that trialkylated organotins are mainly metabolized to their dialkylated forms by hepatic cytochrome P450 enzymes through hydroxylation and dealkylation [29]. Ueno *et al.* showed that inhibition of hepatic cytochrome P450 enzymes prevented TBT-dependent hepatotoxicity [8], suggesting that hepatotoxicity is caused by its metabolites. DBT was hardly degraded in the liver of treated mice, rats and guinea pigs, indicating that the relatively inert DBT may be responsible for some of the TBT toxicity observed *in vivo*.

Glucocorticoids play a crucial role in the regulation of many physiological processes including the control of energy metabolism and the modulation of the immune system. Here, we demonstrate that DBT blocks the glucocorticoid-induced expression of hepatic PEPCK and TAT, two enzymes with a key role in energy metabolism. Furthermore, our results from experiments with LPS-stimulated native human macrophages and human THP-1 macrophages show that DBT is able to abolish the suppressive effect of glucocorticoids on the synthesis of the pro-inflammatory cytokines TNF-α and IL-6. By reducing the GR-dependent trans-repression of NF-κB activation, DBT disrupts the anti-inflammatory effect of glucocorticoids, which plays an important role for the resolution of inflammatory reactions.

Our results indicate that DBT disrupts GR-mediated regulation of gene transcription at the initial step of receptor activation by abolishing ligand binding to the receptor. The effects of DBT on glucocorticoid-induced gene expression in liver cells and in macrophages resembled those of RU486, supporting the evidence that DBT directly inhibits GR activity. The concentrations of DBT that affected GR-mediated gene expression in our experiments were close to those found in tissue and blood samples from human and wildlife in the range of 3–300 nM [5], [30]–[32]. Moreover, we observed a more pronounced effect upon preincubation and after repeated exposure of cells to medium containing DBT. The lipophilic organotin molecules have been shown to accumulate near the lipid-water interface of cellular membranes [33]. Thus, organotins may reach higher concentrations in tissues, especially in those with high lipid content, than in the circulation. A cellular accumulation is also consistent with studies demonstrating up to 70,000 times higher concentrations of organotins in plankton and other organisms compared with sea water [34].

The GR-dependent effects of DBT on the LPS-induced synthesis of TNF-α and IL-6 and the subsequent activation of NF-κB found in the present study are in line with pro-inflammatory effects of DBT and TBT previously reported [35]–[37]. DBT as well as TBT which is metabolized *in vivo* to DBT, induced inflammation of the bile duct associated with hepatic lesions [35] and acute interstitial pancreatitis [36], [37]. The present study suggests that the exposure to DBT can interfere with glucocorticoid-mediated modulation of the immune system and may contribute to inflammatory diseases. DBT and other chemicals disrupting glucocorticoid effects might contribute, among other factors, to the high incidence of allergies and asthma in developed countries [38].

Docking analysis of organotins into the GR 3D-structure revealed that DBT, TBT DPT and TPT dock nicely into a site overlapping the dexamethasone binding site, which would be expected because these organotins are smaller than dexamethasone and should fit into this site. However, DBT and TBT have fewer interactions with the GR than dexamethasone, DPT and TBT. TBT, DPT and TPT did not inhibit GR-mediated transactivation, suggesting that the inhibitory actions of DBT are due to binding to an allosteric site. This hypothesis is supported by DBT inhibition of GR-mediated transcriptional activity in the presence of 100 nM cortisol, which is over ten-fold higher than the K_d_ of cortisol for GR. At this concentration, the steroid binding site on the GR will be occupied by cortisol.

Docking analysis identified a binding site for DBT close to Gln^570^ on α-helix 3 and Arg^611^ on α-helix 5. DBT has several van der Waals contacts with residues in this allosteric site on the GR. Analysis of DBT in this site in the apo-GR and holo-GR reveals that DBT alters the interaction of dexamethasone with the GR. Analysis of the GR with DBT in the apo-GR revealed a conformational change in the steroid binding pocket which results in steric clashes between dexamethasone and Gln^570^ and Gln^642^ (Fig. 8A). Energy minimization of DBT in the holo-GR complex removed these steric clashes. However, after energy minization, Asn^564^ moved and lost its stabilizing contact with the C11 hydroxyl on dexamethasone (Fig 8B). The interaction between α-helix 3 and α-helix 5 is important in transcriptional activity of steroids for the GR [22]. Alteration of α-helix 3 by DBT occupying the allosteric site on the GR may explain how DBT inhibits dexamethasone binding to GR and its subsequent transcriptional activation.

Although neither TPT nor TBT altered GR-mediated transcriptional activity in the absence of cortisol, both of these organotins stimulated the activity of the GR-cortisol complex. Binding of TPT and TBT to other proteins of the transcriptional complex or effects on post-translational modifications of the receptor or its associated proteins could be important in TBT- and TPT-induced stimulation of GR-mediated transcription in the presence of cortisol.

In conclusion, the present study demonstrates that DBT, but not TBT, DPT or TPT, inhibits ligand binding to GR and subsequent stimulation of its transcriptional activity. Molecular modeling analyses indicate that binding to an allosteric site by DBT, but not by the other organotins, alters the orientation of key residues in the ligand binding pocket of the GR. Disruption of GR activation by DBT can disturb essential physiological processes such as the immune system, as shown by inhibition of glucocorticoid-mediated suppression of pro-inflammatory cytokine production in macrophages. Thus, by interfering with GR function, DBT may contribute to immune diseases.

## Materials and Methods

### Analysis of transcriptional activation of reporter genes

HEK-293 cells were cultured in Dulbecco's modified Eagle medium (DMEM), supplemented with 10% fetal calf serum, 4.5 g/L glucose, 50 U/mL penicillin/streptomycin, 2 mM glutamine, and 1 mM HEPES, pH 7.4. 200,000 cells/well were seeded in poly-L-lysine coated 24-well plates, incubated for 16 h and transfected by the calcium-phosphate method with plasmid for human GR-α (0.1 µg/well), MMTV-lacZ β-galactosidase reporter (0.15 µg/well) and pCMV-LUC luciferase transfection control (0.05 µg/well). Cells were washed twice with serum- and steroid-free DMEM 6 h later, followed by incubation for 16 h with organotins. Cortisol (100 nM) was added and cells were incubated for another 20 h. Cells were washed with PBS and lysed with 50 µl lysis buffer (Tropix, Applied Biosystems, Foster City, CA) supplemented with 0.5 mM dithiothreitol. 20 µl of lysate were analyzed for β-galactosidase activity using the Tropix kit and luciferase activity using a home-made luciferine-solution [39].

To measure NF-κB-dependent transcriptional activity, HEK-293 cells were transfected with plasmid 3xMHCLUC (provided by Dr. J. Cidlowski, [40]), containing three binding sites for NF-κB in the promoter preceding a luciferase gene, and a cytomegalovirus (CMV)-driven galactosidase control plasmid to adjust for transfection efficiency. The medium was exchanged by serum- and steroid-free DMEM 6 h post-transfection, and cells were incubated overnight with DBT. Cells were stimulated by adding 5 nM TNF-α with or without glucocorticoids or RU486, followed by incubation for another 24 h. Thereafter, luciferase and galactosidase activities were measured as described above.

### Glucocorticoid receptor binding assay

HEK-293 cells (400,000 cells/well in 12-well plates) were transfected with 0.2 µg/well GR plasmid, washed twice 6 h later with serum- and steroid-free DMEM and incubated in this medium overnight, followed by addition of vehicle (0.1% of methanol or dimethylsulfoxide) or DBT, dexamethasone and 10 nM [^3^H]-dexamethasone (specific activity 70 Ci/mmol). Alternatively, cells were preincubated overnight with vehicle or DBT prior to incubation with dexamethasone. After incubation with dexamethasone for 3 h, cells were washed three times with PBS, lysed with 300 µl of 0.4 N NaOH, and lysates were subjected to scintillation counting. Non-specific binding was determined in cells transfected with pcDNA3 control plasmid or in the presence of 1 µM RU486 and subtracted from the values obtained in absence of antagonist.

### Glucocorticoid receptor expression analysis

HEK-293 cells transfected with GR plasmid were washed 6 h later with serum- and steroid-free medium and incubated in this medium for 16 h with vehicle control (0.1% DMSO), 10 nM dexamethasone or 500 nM DBT. After preincubation, cells were incubated for another 3 h in the presence of vehicle, 10 nM dexamethasone or 500 nM DBT and 10 nM dexamethasone. In a another sample, cells were preincubated overnight in serum-free medium, followed by incubation for 3 h with 500 nM DBT and 10 nM dexamethasone. These conditions were chosen according to the procedure used for GR ligand binding. GR protein was detected by rabbit polyclonal anti-human GR antibody at a working dilution of 1∶500 (Santa Cruz, antibody sc-1030). Actin served as a control to adjust for the amount of protein loaded on the SDS-gel and was detected with a rabbit polyclonal antibody. A horse-radish peroxidase conjugated mouse anti-rabbit antibody was used as secondary antibody for detection.

### Analysis of phosphoenolpyruvate carboxykinase (PEPCK) mRNA levels

Rat H4EII hepatoma cells were cultured in MEM medium containing 1 mM sodium pyruvate and supplemented with 10% fetal calf serum, 4.5 g/L glucose, 50 U/mL penicillin/streptomycin, 2 mM glutamine, and 1 mM HEPES, pH 7.4. H4EII cells were preincubated for 16 h in serum-free MEM in the presence of various concentrations of DBT or vehicle (dimethylsulfoxide), followed by adding 10 nM dexamethasone and incubation for another 24 h at 37°C. PEPCK mRNA expression was determined by real-time RT-PCR as described earlier [41]. Briefly, total mRNA was extracted from H4EII cells (500'000 cells per well) using an mRNA isolation kit, and 100 ng of mRNA was reverse transcribed using SuperscriptII reverse transcriptase according to the manufacturer's protocol (Invitrogen, Carlsbad CA). Relative quantification of PEPCK mRNA expression levels was performed on an ABI7600 Sequence Detection System (Applied Biosystems, Foster City, CA). PCR reactions were performed in 96 well plates using 25 ng of cDNA, TaqMan Universal PCR Master Mix and assay on demand primers and probes from Applied Biosystems (Mm01247057_g1) following the instructions from the manufacturer. The relative expression of each gene compared to the internal control β-actin was determined using the 2-ΔCT method.

### Determination of tyrosine aminotransferase (TAT) activity

H4IIE cells (300,000 cells/well in 6-wells plate) were allowed to adhere overnight, and then washed twice with serum- and steroid-free MEM followed by 16 h incubation with DBT. Dexamethasone (5 nM) was added, and cells were incubated for another 20 h, washed once with PBS and lysed in lysis buffer (50 mM Tris-HCl, pH 7.4, 50 mM NaCl, 0.2% Nonidet-P40) supplemented with Complete Mini Protease Inhibitor cocktail at a working dilution of 1 tablet in 25 ml (Roche Applied Science, Rotkreuz, Switzerland). After centrifugation (10 min, 10'000×g, 4°C), cleared lysates were used to measure TAT activity. 50 µl of lysate (diluted in lysis-buffer) was added to 465 µl of reaction mix (90 mM potassium-phosphate buffer, pH 7.4, 25 mM L-tyrosine disodium, 90 mM α-oxoglutarate, pH 7.0, 0.45 mM pyridoxal-5′-phosphate, pH 6.5) and incubated for 30 min at 37°C. The reaction was stopped by adding 35 µl 10 N KOH, and after 30 min incubation at room-temperature, the absorbance at 331 nm against a blank (lysis buffer treated identically) was determined. The activity was normalized to protein content in cell lysates, as determined by BCA-solution (Pierce, Soccochim, Lausanne, Switzerland).

### Analysis of cytokine production

THP-1 cells and native human monocytes (50,000 cells/well, 24-well plates) were grown in RPMI-1640 medium supplemented with 10% FCS, 50 U/ml penicillin/streptomycin, 2 mM glutamine and 25 mM HEPES, pH 7.4. Peripheral blood mononuclear cells were purified from blood of non-smoking healthy male volunteers of age 25–40 [42]. Venous EDTA-blood (180 ml) was collected and left to sediment in 6% dextran. Leukocytes were enriched for monocytes using a two-step discontinuous Percoll gradient (1.0791 and 1.0695 g/ml). The purity of isolated monocytes was 70–80%. Native human monocytes were differentiated into macrophages by incubation for 7 days in culture medium without medium change, whereas THP-1 cells were differentiated by incubation with 5 ng/ml PMA for 72 h. The medium was replaced by fresh RPMI-1640 containing DBT, and cells were incubated for 16 h. Then, 10 nM dexamethasone and 30 ng/ml LPS were added and cells incubated for another 20 h before harvesting the medium and analysis of IL-6 and TNF-α by ELISA (BD Biosciences, Allschwil, Switzerland).

### Docking of organotins within human GR

The PDB files for DBT, TBT, DPT and TPT were obtained from CambridgeSoft's Chemfinder chemical compound database. Human GR (PDB∶1M2Z) [21] was extracted from the PDB [43] and converted into an apo-monomer using a text editor. Then, organotins were docked into human GR using Autodock 4 [18], [19]. The well depth and equilibrium separation for tin in DBT and other organotins were set to 0.63 kJ/mol and 4.40 Å. Initially, the grid size was set to cover the entire receptor. Lamarckian Genetic Algorithm (LGA) docking was run for 100 trials of 25 million energy evaluations each and a histogram of the binding energies for the various conformations was calculated [19]. This analysis identified two interior sites in the GR. One site overlapped the steroid-binding site. The other site was close to the A ring of dexamethasone. Several non-specific sites for TPT and other organotins were found on the surface of the GR. To analyze docking of organotins to sites with a specific mode of binding, we adjusted the grid to cover the interior of the GR and redid the docking as described above.

The organotin-GR complex calculated by Autodock was refined with Discover 3 software in Insight II, allowing the GR and the organotin to move to an energy minimum. Discover 3 was used with the ESSF force field and a distant dependent dielectric constant of 2 for 10,000 iterations in each energy minimization.

### Statistical analysis

Values are expressed as means±SD. Data were analyzed (and significance assigned) using the ratio *t*-test in the GraphPad Prism 4 software. Data were also subjected to one- or two-way ANOVA using SigmaStat software (Jandel Scientific, San Rafael, CA).
